# Aging and Imaging Assessment of Body Composition: From Fat to Facts

**DOI:** 10.3389/fendo.2019.00861

**Published:** 2020-01-14

**Authors:** Federico Ponti, Aurelia Santoro, Daniele Mercatelli, Chiara Gasperini, Maria Conte, Morena Martucci, Luca Sangiorgi, Claudio Franceschi, Alberto Bazzocchi

**Affiliations:** ^1^Diagnostic and Interventional Radiology, IRCCS Istituto Ortopedico Rizzoli, Bologna, Italy; ^2^Department of Experimental, Diagnostic and Specialty Medicine, Alma Mater Studiorum, University of Bologna, Bologna, Italy; ^3^C.I.G. Interdepartmental Centre “L. Galvani”, Alma Mater Studiorum, University of Bologna, Bologna, Italy; ^4^Department of Medical Genetics and Rare Orthopedic Disease & CLIBI Laboratory, IRCCS, Istituto Ortopedico Rizzoli, Bologna, Italy; ^5^Department of Applied Mathematics, Institute of Information Technology, Mathematics and Mechanics (ITMM), Lobachevsky State University of Nizhny Novgorod-National Research University (UNN), Nizhny Novgorod, Russia

**Keywords:** aging, body composition, fat and lean mass, age-related diseases, imaging techniques

## Abstract

The aging process is characterized by the chronic inflammatory status called “inflammaging”, which shares major molecular and cellular features with the metabolism-induced inflammation called “metaflammation.” Metaflammation is mainly driven by overnutrition and nutrient excess, but other contributing factors are metabolic modifications related to the specific body composition (BC) changes occurring with age. The aging process is indeed characterized by an increase in body total fat mass and a concomitant decrease in lean mass and bone density, that are independent from general and physiological fluctuations in weight and body mass index (BMI). Body adiposity is also re-distributed with age, resulting in a general increase in trunk fat (mainly abdominal fat) and a reduction in appendicular fat (mainly subcutaneous fat). Moreover, the accumulation of fat infiltration in organs such as liver and muscles also increases in elderly, while subcutaneous fat mass tends to decrease. These specific variations in BC are considered risk factors for the major age-related diseases, such as cardiovascular diseases, type 2 diabetes, sarcopenia and osteoporosis, and can predispose to disabilities. Thus, the maintenance of a balance rate of fat, muscle and bone is crucial to preserve metabolic homeostasis and a health status, positively contributing to a successful aging. For this reason, a detailed assessment of BC in elderly is critical and could be an additional preventive personalized strategy for age-related diseases. Despite BMI and other clinical measures, such as waist circumference measurement, waist-hip ratio, underwater weighing and bioelectrical impedance, are widely used as a surrogate measure for body adiposity, they barely reflect the distribution of body fat. Because of the great advantages offered by imaging tools in research and clinics, the attention of clinicians is now moving to powerful imaging techniques such as computed tomography, magnetic resonance imaging, dual-energy X-ray absorptiometry and ultrasound to obtain a more accurate estimation of BC. The aim of this review is to present the state of the art of the imaging techniques that are currently available to measure BC and that can be applied to the study of BC changes in the elderly, outlining advantages and disadvantages of each technique.

## Introduction

The rapid increase of elderly population represents a global health problem (1) together with the concurred increased incidence of age-related diseases, including type 2 diabetes mellitus (T2DM), obesity, metabolic syndrome and cardiovascular diseases (CVD). The new field known as “geroscience” recognized that these chronic pathologies and aging itself share seven interconnected common mechanistic pillars such as epigenetics, adaptation to stress, stem cells and regeneration, proteostasis, macromolecular damage, metabolism and inflammation (2). To date, gerontologists propose to focus on these basic aging mechanisms to successfully counteract all the major age-related diseases, and to slow down the aging process (3). In particular, the chronic low grade inflammatory status occurring during aging, called “inflammaging,” (4, 5) is tightly linked to metabolism, because any impairment of metabolic pathways can fuel inflammation. This metabolism-induced inflammation has been recently named “metaflammation” (6). Modern research aimed at finding potential sources of low-grade inflammation is now focusing on the major molecular and cellular mechanisms such as cellular senescence, mitochondrial dysfunction, activation of the inflammasome (7) and changes in gut microbiota composition, which mostly overlap with metaflammation and inflammaging (8). Several studies showed that metaflammation may trigger obesity-induced insulin resistance, suggesting a causative role in T2DM itself (9) and in diabetes-induced complications (10). Murine models of diabetes and obesity showed that an overexpression of pro-inflammatory molecules in fat tissue occur, which in turn increases insulin sensitivity (11). Several studies also revealed that the adipose tissue is a main source of inflammatory molecules, such as IL-6 and MCP1 (12, 13). Metaflammation is mainly driven by overnutrition and nutrient excess (3, 14), which are present in metabolic diseases, but body composition changes occurring with age can also contribute. Indeed, the aging process is characterized by an increase in body total fat mass and a concomitant decrease in lean mass and bone density, that are independent from general and physiological fluctuations in weight and body mass index (BMI) (15). Moreover, the accumulation of muscle fat, visceral fat and liver fat, in form of lipid droplets (LD), also shows an age-dependent increase, while an opposite tendency is observed for subcutaneous fat mass (16). However, it should be taken into account that when the decrease of subcutaneous peripheral fat becomes excessive, it is associated with a pro-inflammatory status, and a reduction of LDs is associated with lipotoxicity (17) leading to CVD, an increased risk of insulin resistance and T2DM. Thus, in order to preserve a metabolic homeostasis and a health status positively contributing to longevity, it is desirable to maintain a balanced rate of fat content and distribution (18). For this reason, a detailed assessment of body composition (BC) in elderly is critical and could be an additional preventive personalized strategy for age-related diseases. The most commonly used method to investigate BC employs a five-level model, which make it possible to classify the human body according to five levels of increasing complexity: I, atomic; II, molecular; III, cellular; IV, tissue-organ; V, whole body. To date, whole-body, organ-tissue, and molecular levels are the most studied in human BC assessment. BMI measurement is widely used as a surrogate measure for body fatness, due to the simplicity of anthropometric methods and the widespread availability of techniques to assess it, however it does not reflect the precise distribution of body fat. A hierarchical cluster analysis based on BMI together with BC parameters revealed that clusters with very similar BMI have a different amount of fat, lean and bone masses (19). Numerous clinical methods and techniques such as waist circumference measurement, waist-hip ratio, underwater weighing and bioelectrical impedance analyses are also available. However, the attention of clinicians has recently focused to several imaging techniques to study BC, because of the great advantages offered by imaging tools in the research and clinical aspects of this field (20, 21). The imaging methods used to analyze BC aim to divide body mass into its components based on their different physical properties. Depending on the information sought, several methods can be used to measure BC, such as computed tomography, magnetic resonance imaging, dual-energy X-ray absorptiometry and ultrasound, each showing specific advantages and limitations. In selecting the diagnostic imaging method, one should consider the accuracy and type of the information obtained, the safety of assessment (*e.g*. in terms of radiation exposure), the time required and costs (equipment and personnel) (Tables 1, 2). Nowadays, Dual-energy X-ray Absorptiometry (DXA) represents a reference method for the assessment of human BC in the research field (22, 23), due to its fast acquisition time, low radiation exposure and relatively low cost when compared to other available techniques (24–27). Within the framework of the European NU-AGE project (conducted from 2011 to 2016) a DXA scan has been carried out in a large number (*N* = 1,121) of sex-balanced, free-living, apparently healthy older adults aged 65–79 years enrolled in 5 European countries (Italy, France, United Kingdom, Netherlands and Poland) (28) for the first time. The results showed that BC characteristics are different in elderly women and men across Europe (19) and that a better adipose-related inflammatory profile is associated to a more favorable BC in terms of fat and lean mass markers (29). In this review, we summarize the present knowledge of available imaging methods to measure BC, with a focus on the measurement of BC changes occurring with age and we discuss *pros* and *cons* of each technique.

**Table 1:** Overview of body composition methods for assessing adiposity and regional fat depots in older adults.

		**Body fat compartment**							
**Method**	**Frequently used measures**	**Visceral fat**	**Inter-/intramuscolar fat**	**Whole body fat**	**Low cost**	**Availability**	**Radiation exposure**	**Precision**	**Accuracy**
Anthropometry	Body Mass Index	+	+	++	+++	+++	+++	+	++
	Skinfold thickness	+	+	++	+++	+++	+++	+	+
	Waist circumference	++	+	++	+++	+++	+++	+	+
	Arm circumference	+	+	+	+++	+++	+++	+	++
	Predicted fat mass	+	+	++	+++	+++	+++	+	+
Bioelectrical impedance	Predicted fat mass	+	+	++	+++	+++	+++	+	+++
Ultrasound	Mid-tight image	(+)++	++	+	+++	+++	+++	++	+++
Dual energy X-ray absorptiometry	Whole body scan	++	+	+++	++	++	++	+++	+++
Computed tomography	Abdominal image	+++	++	+	+	+	+	+++	++
	Mid-tight image	+	+++	+	+	+	+	+++	+++
Magnetic resonance imaging	Abdominal image	+++	++	+	+	+	+++	+++	++
	Mid-tight image	+	+++	+	+	+	+++	+++	+++
	Total body multi image	+++	+++	+++	+	+	+++	+++	++

*+++ indicates a very positive feature of the method, while + indicates a less positive feature*.

**Table 2:** Overview of body composition methods for assessing whole body and regional skeletal muscle in older adults.

		**Skeletal muscle compartment**						
**Method**	**Frequently used measures**	**Regional muscle**	**Whole body muscle**	**Low cost**	**Availability**	**Radiation exposure**	**Precision**	**Accuracy**
Anthropometry	Arm circumference	++	+	+++	+++	+++	+	+
	Calf circumference	++	+	+++	+++	+++	+	+
	Predicted ASMM	+	++	+++	+++	+++	+	+
Bioelectrical impedance	Predicted FFM mass	+	+	+++	+++	+++	+	+++
	Predicted ASMM	++	++	+++	+++	+++	+	+++
Ultrasound	Mid-tight image	++	+	+++	+++	+++	++	+++
Dual energy X-ray absorptiometry	Whole body scan	+++	++	++	++	++	(+)++	+++
Computed tomography	Mid-tight image	+++	+	+	+	+	+++	+++
Magnetic resonance imaging	Mid-tight image	+++	+	+	+	+++	+++	+++
	Total body multi image	+++	+++	+	+	+++	+++	++

*ASMM, Appendicular Skeletal Muscle Mass; FFM, Fat-Free Mass*.

*+++ indicates a very positive feature of the method, while + indicates a less positive feature*.

## Dual-Energy X-ray Absorptiometry (DXA)

DXA was originally developed to evaluate bone mineral density, but it has gained popularity as a method to assess whole-body and regional soft-tissue composition. DXA divides the body into three components; bone mineral content (BMC), lean mass (LM) and fat mass (FM). Since the method assesses three body-composition components at a molecular level, it is widely considered as the gold standard for BC assessment in clinical practice.

This technique is based on the physical principle that X-rays of different energies are differentially attenuated when passing through the various tissues of human body. By radiating the body in anterior-posterior direction using two different energies, and assuming a two-compartment model in each measurement point (pixel), the image can be reconstructed; the two-compartment model assumes that pixels not containing bone depend on LM and FM ratio, and that pixels containing bone depend on BMC and soft tissue ratio with a subsequent interpolation of LM and FM ratio based on neighboring pixels not containing bone.

**Figure 1:**
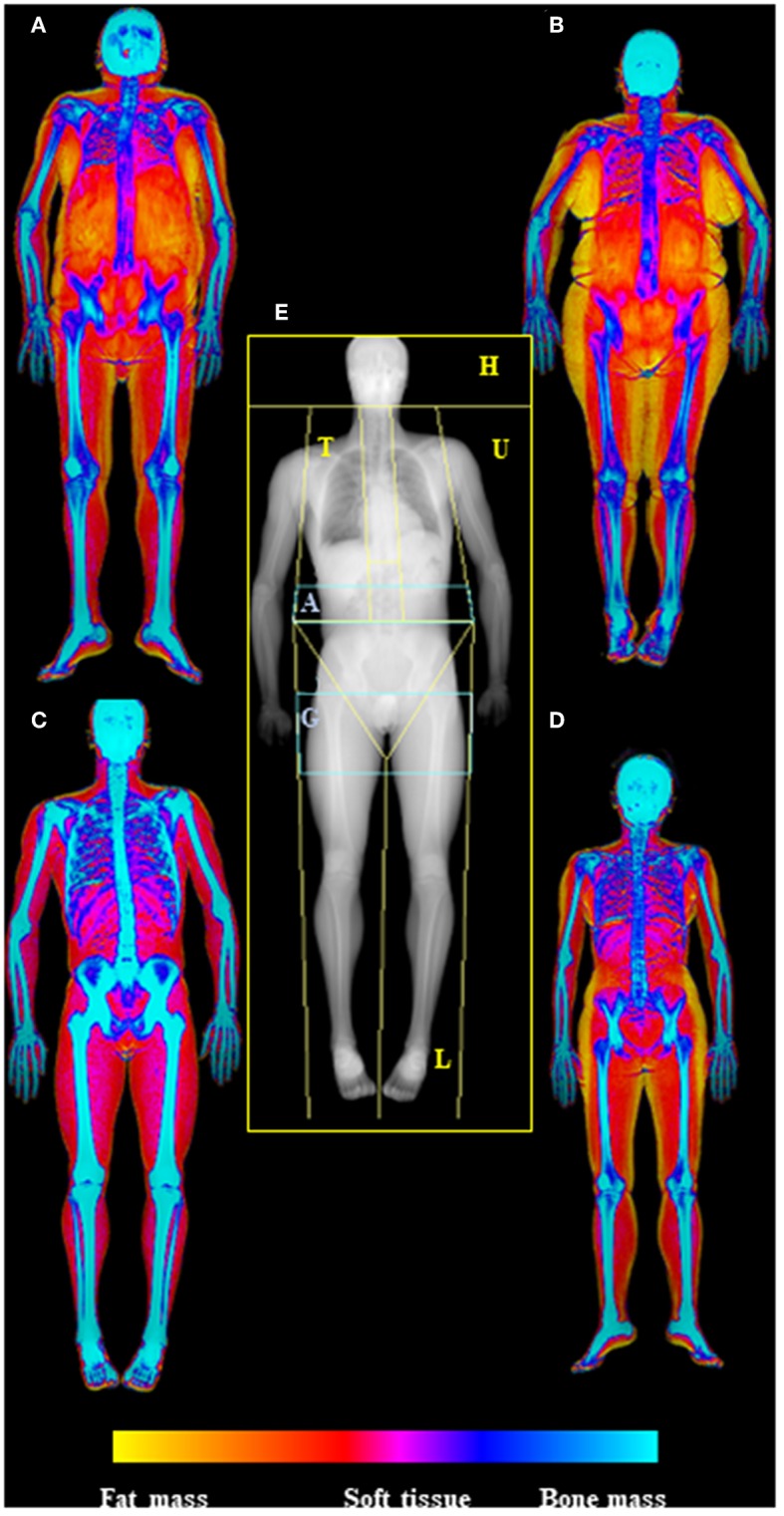
Dual-energy X-Ray absorptiometry (DXA) examination of body composition. In the center of the picture **(E)** is represented the skeletal map of whole body scan by DXA highlighting the standard ROIs specifics for body composition assessment (head—H, trunk—T, upper limbs—U, lower limbs—L), with the two regions at “high metabolic significance” representing by gynoid (G) and android (A) regions. On the side are depicted the soft tissue maps of whole body DXA scan (from fat mass—yellow—to bone mass—blue); in particular on the left are visualized old **(A)** and young **(C)** males (respectively upper and lower), while in the right old **(B)** and young **(D)** females (respectively upper and lower), highlighting the increase of fat mass in aging. Images are kindly provided by IRCCS Rizzoli Orthopedic Institute, Unit of Diagnostic and Interventional Radiology (2019).

DXA allows total-body and standard regional body composition measures, including trunk, arms, legs, android and gynoid regions, and ideally, can estimate every human body part of interest (Figure 1).

In addition, it is now possible to estimate with DXA the amount of visceral adipose tissue (VAT) and subcutaneous adipose tissue (SAT) in the android region that represent, a harmful factor and a presumable protective factor, respectively, in cardio-metabolic status of patient.

The DXA approach represents a good candidate to be a gold standard technique to measure longitudinal changes in BC in multiple pathologic or paraphysiologic conditions because of its high accuracy and precision, large availability, and low-cost.

DXA is a non-invasive, quick and safe method for BC assessment, and the radiation exposure is considered small and safe for repeated measures (a whole-body scan takes only 6–10 min and the radiation exposure is equivalent to a day spent sunbathing).

Currently available DXA systems for scanning whole-body tissue composition are capable to analyze a wide range of weights and sizes, including severe obese subjects (>200 kg with relatively wide scanning space >65 cm).

A marked impaired hydration status may affect DXA accuracy because of the programmed assumption of a constant and uniform LM hydration (25).

Reference values of BC assessed by DXA on adults over 60 years old are available from the National Health and Nutrition Examination Survey 1999–2004 and from other studies on local population (30).

## Ultrasound (US)

Ultrasound is another technique that has been used for a long time to assess FM. US, based on echo reflections, offers a two-dimensional gray-scale image, between black (no echoes) and white (strong reflections), and shows skin-subcutaneous fat borders, fat-muscle, and muscle-bone interfaces (31). Although US procedures are considered accurate, reproducible, and fast for the analysis of abdominal adiposity by allowing a local, easy and close-at-hand evaluation of subcutaneous and visceral fat compartments (32), there are different opinions about its validity. Borkan et al. suggested that with respect of ultrasound, skinfolds were a better measure of subcutaneous fat (33), while Fanelli and Kuczmarski proposed that US was identical to skinfolds when determining body fat (34). US intra-abdominal thickness measurement was introduced by Armellini and colleagues to demonstrate that US was the most powerful identifier of visceral adipose tissue area into intra-abdominal thickness (35, 36).

It is easy to understand how the absence of a straight standardized protocol leads to a decrease in accuracy and reliability of US measurements of BC, mainly for visceral adiposity. In a recent study was demonstrated that reproducibility and repeatability, especially for visceral fat, were proved more stable in fasting state and expiration (37).

## Computed Tomography (CT) and Magnetic Resonance Imaging (MRI)

CT and MRI are cross-sectional imaging modalities providing 2D or 3D maps of pixels allowing for the *in vivo* measurement of lean mass and total adipose tissue and its subdepots (subcutaneous, intermuscular, and visceral).

**Figure 2:**
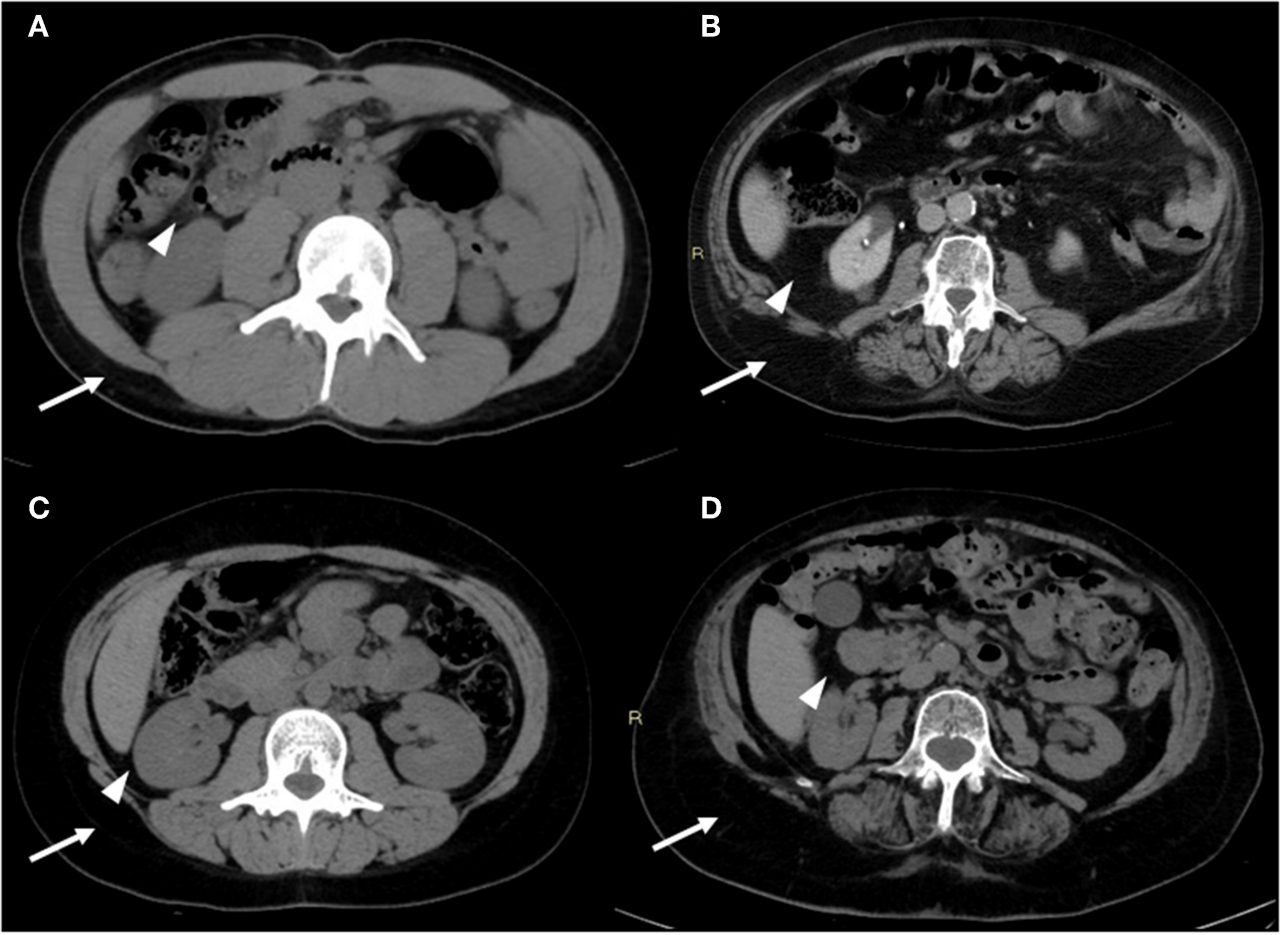
CT images of the android region. CT image slices of the android region showing changes in adiposity distribution (visceral fat—arrowheads; subcutaneous fat—arrows) depending on age and sex: **(A)** young male, **(B)** old male, **(C)** young female, **(D)** old female. With advancing age, there is a redistribution of fat mass compartment with increase of visceral compartment for both sexes (in particular for males); it is also noteworthy that the subcutaneous compartment is prevalent in females, both in young and old age. Images are kindly provided by IRCCS Rizzoli Orthopedic Institute, Unit of Diagnostic and Interventional Radiology (2019).

CT presents great practical significance due to its routinely use for diagnosis and follow-up in various diseases and allows an accurate quantification of whole-body composition (Figure 2).

Being a volumetric technique, CT allows to measure body components at tissue-level using pre-established Hounsfield Units (HU) to recognize different tissue density (soft tissue: 30–50 HU; fluid-sovrafluid: 0–30 HU; adipose tissue: −100 HU; bone and calcification: 100–1000 HU).

CT imaging at L3 level provides total, visceral or subcutaneous adipose fat area, visceral adipose volume, total psoas area, and skeletal muscle index (SMI) (38). Moreover, according to ethnicity- and sex-specific data, CT has been used to derive a predictive cardio-metabolic risk (CMR) equation (39). This type of evidence endorsed other specific research, analyzing pericardial fat, intrathoracic fat and epicardial fat, showing the potential contribution in CMR stratification (40). Also, because CT images targeted on the III lumbar vertebra are similar to those on chest, they could be tentatively performed solely. As CT usage has now increased in clinical practice, the radiation exposure should be taken in mind, since it represents a risk factor for oncologic disease development.

**Figure 3:**
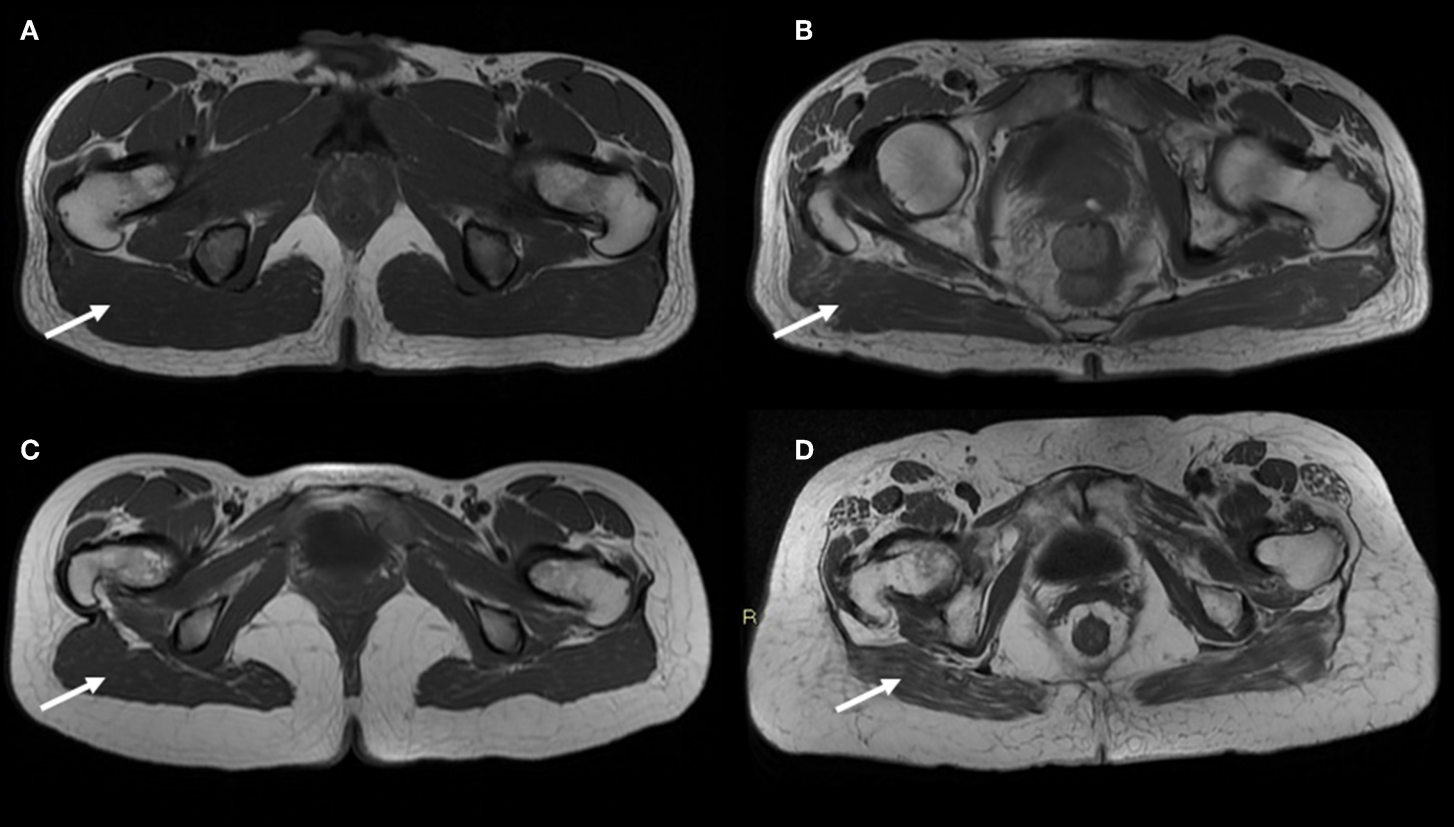
MR T1-weighted image slices of the gynoid region showing age-related muscle changes in both sexes (poor muscle quality and fat infiltration—arrows). In addition larger subcutaneous adipose tissue are observed in the gynoid region of an old female **(D)** compared to a young female **(C)**; on the contrary the representation of subcutaneous compartment in the same region is the same both for a young male **(A)** and an old male **(B)**. Images are kindly provided by IRCCS Rizzoli Orthopedic Institute, Unit of Diagnostic and Interventional Radiology (2019).

Differently from CT that is calibrated against the Hounsfield scale, signal intensities in MRI are often non-quantitative because image intensity values do not reflect physical properties of the imaged body. MRI allows to measure body fat-free mass such as skeletal muscle mass at arms, legs and trunk level, specific organ masses, and provides also an estimate of bone marrow adipose tissue (41). From a technical point of view body composition measurement with MRI is based on the different magnetic properties of hydrogen nuclei contained in water and fat. Several MRI sequences have been developed to measure body fat, using variations in radiofrequency pulse to differentiate between adipose tissue and fat-free mass (27). A variety of pulse sequences are thus available to generate contrast between fat and non-adipose tissue (42). Adipose tissue is characterized by a short T1 and a long T2 relaxation time; in T1-weighted spin-echo sequence, fat appears as a high signal (white) because of a high concentration of relative immobile protons, thus differentiating it from muscles, fluids, bone and internal organs, which appear as gray signals (43). The time of acquisition for such sequences is relatively long and implies some issues, such as respiratory/motion artifacts. Variations of this sequence have been developed in order to reduce the acquisition time. Nowadays, a whole-body MRI scan of an individual can be obtained in about 5 min, allowing for the detailed evaluation of total and regional fat depots. Whole-body scanning is the most accurate and reproducible protocol to obtain an accurate quantitative map of body fat distribution and content, but it has been mainly limited to research studies due to the high scan costs and the need of time-consuming image analysis (44). In fact, the amount of data generated by whole-body MRI requires a complex analysis, generally not manually feasible, except for very small studies. In the last years, this has led to the development of semiautomated or automated methods for MRI-based body composition analysis. Furthermore, single-slice and region-specific multi-slice protocols were developed to make data analysis easier and faster (Figure 3) (43, 45). An alternative to whole-body imaging is the acquisition of the solely abdominal region, which allows to measure fat depots frequently associated with CMR factors, like visceral adiposity (46). Multi-slice protocols have become the preferred method for large population studies, while single-slice protocols have been mainly used in small cohort studies, even if a number of protocols differ in the landmarks to be used for acquisition; the level of L4–L5 has been the most commonly reported anatomical landmark for single-slice imaging, while a level close to L2–L3 has been considered by several authors as the preferable site to evaluate visceral adipose tissue depot (41, 43). A poor prediction of visceral and subcutaneous tissue changes was reported in a longitudinal study with single-slice MRI evaluation at L4–L5 level (47).

There is an increasing interest in using MRI to evaluate age-related muscle changes to understand the contribution of poor muscle quality and fat infiltration in sarcopenia. Recently, Yang et al. demonstrated that a single slice cross-sectional area at mid-femur can be used in clinical practice for a fast and non-invasive diagnosis of sarcopenia in old adults (48). Compared to other imaging techniques, a key advantage of MRI is the ability to detect changes in the muscle structure occurring during the aging process or during disease progression, making this technique a powerful tool in longitudinal studies. Quantitative magnetic resonance imaging (QMRI) can be achieved by proton nuclear magnetic spectroscopy or magnetic resonance spectroscopy (MRS), which allows the accurate measurement of intramyocellular lipid and extramyocellular lipid in muscle fibers. MRS can precisely discriminate adipose and lean tissue by enhancing contrast, offering the possibility to estimate the accumulation of tryglicerides in non-adipose tissue (ectopic lipid). Diffuse fat infiltration in organs and lean tissue can be also estimated using “quantitative fat-water imaging,” which is based on Dixon imaging, a gradient recalled echo imaging method which uses the chemical shift between proton resonance frequencies in water and in fat (44). MRI shows the best contrast between fat and muscle tissue, allowing for an accurate evaluation of muscle quality. It has been shown to possess a higher sensitivity compared to CT in detecting early fatty replacement in muscles (49). Differently from DXA, QMRI has the great advantage to be independent of fat-free mass hydration level, showing great accuracy and low-minimal changes detectable in longitudinal studies. However, underestimation of fat mass and overestimation of fat-free mass by QMRI compared with a 4-compartments model has been reported (50). In old adults infiltration of adipose tissue is recognized as a predictor of poor muscle and mobility functions. MRI was used to study intramuscular adipose tissue in frail and non-frail individuals, showing that more muscle fat infiltration was detectable in older frail subjects (18.0 vs. 11.7%) (51). In women over 50 years old, MRI-measured muscle fat infiltration was reported to be positively associated with increased fracture risk (52), while lower extremity muscle fat infiltration was shown to be negatively associated with performance based measures of physical function (53).

Currently, MRI represents the most advanced and accurate technique for the study of body composition, by allowing the measurement and quality assessment of muscle volume and cross-sectional analysis. Its ability to detect changes in the muscle structure occurring with aging makes this technique extremely fascinating to understand age-related progressive loss of muscle strength and quality. MRI, together with CT, represents the gold-standard technique in exploring muscle mass and quality for research purpose, however the limited access to the equipment, the complexity of data analysis and high cost, limit the use of MRI routinely in clinical practice (54). A strong methodological weakness is represented by the lack of a standardized evaluation protocol in image analysis, limiting comparison between studies (55).

## Changes in Body Composition With Age

### Overall Adiposity

From young age to about 75 years old, body weight and consequently BMI usually increases. This trend is followed by a decline with an intermediate period of stability (56). Due to physiologic height loss with aging, the BMI in elderly may be overestimated in weight-stable persons and this condition is particularly true in women >85 years old (57). In elderly, recurrent weight changes events are usual and the individual evolutions of body weight and BMI are very miscellaneous (58, 59).

In a healthy Italian population, an increase of FM and a decrease of LM were detected up to 70 years old, although women were less affected by this phenomenon. As the matter of fact FM in women increased up to the first four decades of life and remained steady afterwards, differently from the development of FM in men (remarkable increase, especially after 60 years old) (22).

Consequently, the percentage of body fat in both sexes increases up to ~70 years old, but these percentage modifications appear less evident later, because of a trend to a reduction in fat mass after 80 years old (60). In weight stable old adults, the loss of skeletal muscle mass contributes to an increase in body fat percentage (61, 62).

The complex and partially unknown endocrine role of adipose tissue on muscle metabolism emphasizes the existence of an adipo-muscular axis that can influence metabolic changes between physiologic and pathologic states, including obesity and inflammation, but also in para-physiologic conditions such as aging. As examples, an accelerated loss of LM has been associated with greater body fatness in old age and a significantly greater quantity of LM is lost during weight loss than is gained during weight increase, specifically in old men (63, 64).

### Body Fat Distribution

A general increase in trunk fat (largely visceral fat) and a decrease in appendicular fat (largely subcutaneous fat) have been observed with age. The reduction in subcutaneous adipose tissues has also been confirmed using whole-body MRI as well as CT data of the mid-thigh or US measurements at abdominal level. The increase in abdominal adiposity has been estimated through anthropometric methods such as waist circumference, but a direct measurement can be obtained only by imaging techniques such as CT and MRI (expensive but accurate) as well as US and DXA (available but less accurate).

In a recent study evaluating adiposity markers of visceral fat by US, no differences were found between males and females in their 30 to 50 s, while a significant difference emerged for those in their 60 to 70 s; the visceral fat content increases noticeably during aging in males, while in females major changes were significantly observed in the preperitoneal circumference (65).

Considering DXA imaging analysis, a significant redistribution of both central and visceral FM was shown to happen for men during their lifetime, but not for women. In a healthy sample, android and visceral fat progressively increased in elderly males, while females, in old decades, seem to go toward a less pronounced android or visceral redistribution of fat. While in healthy females the central FM distribution maintains a stable android/gynoid ratio, Bazzocchi et al. showed that in males a linear progression to an abdominal redistribution of FM could be observed over time. In particular, SAT depots were significantly higher in females, becoming nearly overlapping in males and females from 50 to 70 years old (22).

Apart from the redistribution of body fat, another feature of old age-related BC changes are the accumulation of fat infiltration into non-fat tissues. The alterations in ectopic fat have been mostly studied in aging muscles, especially with the support of whole-body MRI scans. Several evidences in literature suggests that the amount of inter-muscular adipose tissue increases rapidly with aging: about +10% and +6% per year in old men and women, respectively. In particular, the increase in inter-muscular adipose tissue is most visible in those who underwent an increase in total body weight, but it also accumulates in people who experience weight loss (66–68).

### Skeletal Muscle Mass

In 1997, the age-related loss of muscle mass was termed sarcopenia, from the Greek words *sarx* (meaning flesh) and *penia* (meaning loss). Forbes and Reina were among the first to report prospective data that showed an age-related decrease in lean body mass (about −0.41 kg per year in adults).

However, it is globally accepted today that the concepts of low lean mass and decreased muscle function should necessarily be both incorporated into a current definition of sarcopenia.

According to this tendency, several working groups worldwide have new consensus definitions of sarcopenia published in recent years, even if a unique consensus with regard to the specific cut-off point or the most appropriate technique for assessment of low skeletal muscle mass in old adults has not been identified yet (23, 69–71).

In some studies, the deterioration in skeletal muscle mass in elderly have been measured by using 24-h urinary creatinine excretion and CT cross-sectional area, providing an accurate assessment of the skeletal muscle mass loss because other lean tissues, in particular bone and visceral organs, are excluded from muscle evaluation. From these findings, the relative yearly decline in the skeletal muscle mass was evaluated to be between −0.64 and −1.29% per year for old men, and between −0.53 and −0.84% per year for old women (63, 66, 67, 72–74). Both the increase in body fat and the loss of muscle mass with age make old adults at a higher risk of developing sarcopenic obesity, a condition characterized by excess of body adiposity associated with a reduced muscle mass and/or strength (75, 76).

More recent studies using DXA showed a general decrease of LM at upper and lower limbs with age in both sexes. Considering FM/LM distribution at the appendicular body, the decrease of LM was associated to an increase of FM. In particular, LM impoverishment was reported after 40 years in men (remarkably after 50 years old), and later, in the 50 years old, in women. Moreover, women seemed to maintain a more favorable arm masses ratio during aging. In this study, anthropometry was reported to be scarcely representative of LM of arms in both genders, independently of age, therefore the authors suggested that a correct assessment of BC at limbs should be achieved by imaging such as DXA (22, 77).

## Associations of Fat Mass With Mobility, Disability, and Mortality

In the elderly, obesity determined by a high BMI has been shown to be tightly associated with a decline in functional performances, possibly leading to disability. For example, a prospective study from Koster et al. involving almost 3,000 participants between 70 and 79 years old showed that a BMI above or equal 30 kg/m^2^ was associated with a 60% increased risk of mobility limitations, which was reported to be independent of the participants lifestyle habits. This finding is consistent with the idea that obesity could be an important factor affecting the functional status of individuals rather than a mere indicator of physical inactivity (78).

It is not clear if an increased risk of functional limitations in the elderly is also associated with overweight, i.e., a BMI comprised between 25.0 and 29.9 kg/m^2^. A study involving 406 participants aged 70–89 years showed that the risk of developing major mobility limitations was reduced in overweight individuals compared to normal weight or obese subjects. However, several studies indicated that a high abundance of body adiposity in the elderly may lead to an increased risk of mobility limitations and disability (79–85).

Adiposity is not the only determinant of functional status in old age; individual lifetime histories of being overweight or obese is also to be considered when considering the risk of disability. It has been reported that in older men and women who have been overweight or obese since age 25, the risk of developing mobility limitations was almost 3 times higher compared to individuals which maintained a normal weight throughout their lifetime. Conversely, individuals who became overweight or obese only in old age showed a risk 1.7 times higher. Thus, a longer history of high body fatness appears to augment the risk of functional failure in old age. Weight gain is another significant determinant of functional performances in advancing age, as suggested by several prospective studies. For example, in a cohort of almost 3,000 Italian individuals over 65 years old, a weight gain of more than 5% after their 50s was correlated with an augmented risk of limitations in activities of daily living (ADLs) (86–89).

However, it was also observed that a 7-years weight gain pattern among men and women over 65 years old did not increase the risk of limitation in ADL or mobility compared to individual who maintained a stable weight (59).

Weight instability and oscillations have been associated with a higher risk of limitation of ADL and mobility infirmity in the elderly (59, 79, 90).

Most of the weight changes reported in these studies were unintentional. In other intervention studies, improvements of physical performances in obese old adults after intentional weight loss following dietary restriction were reported. Thus, the American Society for Nutrition has recognized the functional benefits of intentional weight loss in obese elderly (91–93); nevertheless, additional studies are required to set up optimal weight loss strategies for obese older adults and to assess their long-term benefits.

Generally, the correlations between BMI and mortality in the elderly have been described showing U-shaped or J-shaped relationships. An increased risk of death is associated with a low BMI (underweight), although a possible causal relationship linking an underlying illness (for example cancer) causing a low BMI and the consequent increased mortality rate cannot be excluded. An increased mortality risk has been sometimes reported only for obese elderly, while others indicate an increased mortality risk in overweight old adults. Therefore, a clear relationship is still a matter of debate (94–96).

Surprisingly, in some observational studies a protective effect of high levels of body fatness on mortality have been reported in the elderly (97). However, these studies are potentially inconsistent due to methodological biases in sampling and statistical analysis that may increase the reported protective effects on mortality. In fact, other studies not suffering from sampling or grouping biases conclude that being either overweight or obese decrease the chance of a healthy aging. A J-shaped association between BMI and 10-years mortality was detected among non-smokers older adults (98). A systematic review and meta-analysis examining the impact of a high BMI on mortality risk in older adults concluded that BMI in the overweight range is not associated with an increased risk of mortality, whereas obesity showed a significant association with a higher mortality risk. More recent studies have supported the finding that a high BMI negatively affects healthy life expectancy, and it is also associated with an increased risk for cancer mortality, in particular for colorectal cancer. A difference between men and women exists in the degree that excess body weight increases mortality risk (99–106).

Limiting the analysis to very old adults only, obesity appears to be unrelated to mortality risk and no protective effect of adiposity was observed (107, 108). It is possible that the relationship between adiposity and mortality can assume different meanings depending on the age, and that in some circumstances a higher BMI may be protective, even though more studies are required to gain more insights into this relationship. However, most of the studies conducted so far consider only the BMI as a measure of adiposity. Since the complexity of body fatness cannot be completely explained using the sole BMI, some studies investigated the relationship between mortality risk and adiposity by assessing the impact of different fat compartments. Even these studies resulted in conflicting evidences (102, 109–112).

Another important predictor of mortality risk in old adults is represented by body weight change. In particular, a recent study considering a multiethnic cohort of 63,040 individuals showed that weight loss rather than gain was associated with an increased mortality risk (59, 113–118).

As discussed above, these results suggest that unintentional weight loss may increase mortality risk in older adults, but not intentional weight loss. Unintentional weight loss may be the consequence of an underlying disease. Unfortunately, in most of the studies conducted so far intentional rather than unintentional weight loss distinction is not very clear. Body weight increase has not been found to be associated with a higher mortality risk in older adults (59, 114).

However, using a reliable BC measurement approach, researchers showed that elderly men who gained >5% fat mass over a 4.6-years follow-up had a higher mortality risk compared to men who did not change their fat mass (116). Since weight gain may be the result of an increase in fat as well as muscle mass, which can have a different impact on the associated risks, it is necessary for upcoming studies to evaluate the actual changes in different body compartments to consider their effects on mortality risk. A large waist circumference has been associated with mobility limitations and disabilities in several studies (119–123). In prospective studies, a high-risk waist circumference at baseline (of ~ > 102 cm in men and >88 cm in women) was correlated with a higher incidence of mobility and functional limitations, with a greater association in inactive older adults (78, 124–130). A longitudinal study assessing a 5.5-years modification in waist circumference showed that this was not associated with a change in the self-reported disability, reporting that the main predictor associated with physical decline was indeed the reduction in appendicular fat-free mass (74).

Muscle quality and muscle fat infiltration assessed by CT was associated with a higher risk of incident mobility limitations in men and women over 70 years old (84, 131–133).

High waist circumference in old adults is also a predictor of mortality. Increased mortality risk was observed also in normal BMI individuals who showed a large waist circumference, even if this association was reported to be dependent on cardiorespiratory fitness. It is possible that especially in older men, waist circumference could be a stronger predictor for mortality risk than BMI itself. In fact, in a study evaluating the associations between BMI, waist circumference and specific causes of mortality (such as deaths from lung cancer and chronic respiratory disease), waist circumference but not BMI showed statistically significant positive associations with deaths from major specific causes (102, 110, 134–138). In contrast, it has been shown a protective effect of a larger waist circumference in adults of 65 years old (100, 108).

Some studies suggest a negative impact of abdominal fat in life expectancy of old adults. A J-shaped relationship between DXA-assessed central adiposity and mortality was described (112). Similarly, visceral adiposity determined by CT was shown to be correlated with an increased risk in men over 50 years old (111, 139). A recent analysis investigating the relationship between BC and inflammation in a large European cohort of elderly has shown that BC and regional lean/fat distribution can define BC clusters that differently associate with inflammatory markers and inflammatory profiles (29).

## Associations of Lean Mass With Mobility, Disability, and Mortality

**Figure 4:**
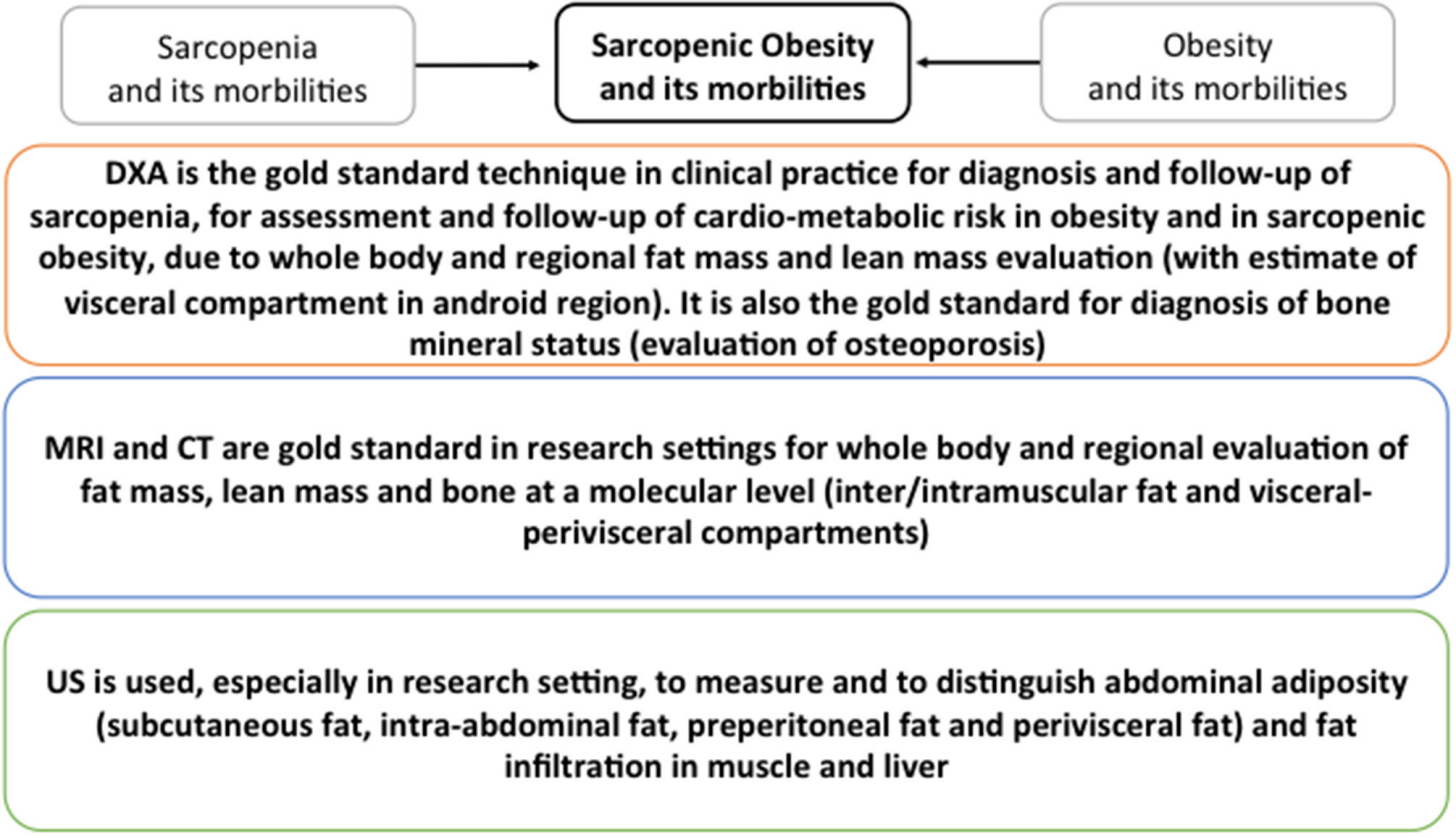
Graphical summary of the standards for DXA, CT, MRI and US for the detection of metabolic dysfunction in elderly. Standards for sarcopenia, obesity and sarcopenic obesity are summarized.

It has been suggested that skeletal muscle mass reduction, or sarcopenia, that occurs in aging is associated with a decline in functional status in the elderly (70). Indeed, several studies have shown that sarcopenia is related to a poorer functional status or to a 5-years functional decline in old age. Surprisingly, two studies have shown that muscle mass gain rather than loss may lead to a worse functional status or greater functional decay. However, this may be due to the interfering role of excess adiposity, which is associated with a higher skeletal muscle mass but a poor functional status, thus the importance of considering the role of body fatness when investigating the correlations between skeletal muscle mass and functional status changes in the elderly (139–144). Several studies have shown that sarcopenia is not associated with or only weakly associated with: (A) a compromised functional capacity (145–149) and (B) a future functional decline (84, 85). According to these studies, which performed careful adjustments for both body fat and body height, the high body fat mass strongly affects functioning in old men and women, regardless of the physical activity level of the participants. This suggests that the impact of an excess body fat on the functional status in old age is far more important than a low skeletal muscle mass. In 2004, the concept of sarcopenic obesity (defined as having a body fat percentage >40% and a skeletal muscle index <5.45 kg/m^2^) was launched, following the results of a study that showed a twofold higher risk of developing instrumental ADL disability in old sarcopenic adults (based on a threshold amount for the appendicular skeletal muscle mass divided by the body height squared) who had a high proportion of body fat compared to elderly with normal fat levels and without sarcopenia. However, more recent cross-sectional studies have not supported the finding that a mixture of low muscle mass and high body fat mass is more disadvantageous to the functional status than a high body fat mass alone. Considering sarcopenia alone, no association with an increased risk of a poor functional status was observed. One other recent study conducted on French women showed that compared to obese women, the sarcopenic obese women tended to have a higher risk of difficulty descending stairs but no differences were found for the other six physical function elements investigated in the study (148, 150–153). According to the present literature, is not possible to convey that the combination of obesity and sarcopenia is more damaging for physical performance than obesity alone. Additionally, it remains unclear whether the risks associated with sarcopenic obesity are higher than the sum of the single risks of obesity and sarcopenia together. The evaluation of body masses by the imaging methods described in this review could support clinical practice for the diagnosis of metabolic dysfunctions in elderly. In particular, because DXA scan can evaluate total and regional fat, lean and bone masses with accuracy for the visceral adiposity in the android compartment, it represents the gold standard for the diagnosis of sarcopenia, osteoporosis as well as of the CMR in both obesity and sarcopenic obesity. MRI, CT, and US are mainly used in research settings. MRI and CT are gold standards for the evaluation of inter/intramuscular fat and visceral-perivisceral compartments particularly important in sarcopenic obesity diagnosis. US is mainly used to measure subcutaneous, peritoneal and visceral fat thus being of particular use for the diagnosis of CMR risk in obesity. The graphical summary reported in Figure 4 shows the standards for each technique.

A clear association between low muscle mass and functional decline in elderly has not been assessed due to the lack of evidences, although it has been suggested that a marked skeletal muscle mass waste in old age might intensify the chance of functional limitations and disability. As an example, a study involving 159 elderly (both males and females) who were monitored up for 5.5 years, showed that the loss of the appendicular muscle mass and leg muscle mass (as measured by DXA) was correlated with a decline of the disability score (74). Changes in the appendicular skeletal muscle mass over 5 years had a faint and positive association with changes in physical functioning measures (144). Unfortunately, it remains not clear whether the actual shrinkage in skeletal muscle mass or the involuntary decrease of body weight, which in turn leads to a decrease in skeletal muscle mass, could be the crucial factor inducing the functional status decline occurring with age. A recent trial showed that after voluntary weight loss, the loss of fat mass in the abdomen and thighs compared to the changes in skeletal muscle mass was the main determinant of improved functional performance (154, 155).

Only three prospective studies conducted so far evaluated skeletal muscle mass in an accurate and precise way and investigated the association between sarcopenia and mortality in older adults. The Health, Aging and Body Composition Study showed that the low muscle mass in the inferior limbs (as assessed by CT or DXA) was not strongly associated with a 4.9-years mortality risk in males and females aged 70–79 years (156). While in men, the low midthigh muscle cross-sectional region (as measured by CT) was associated with mortality (HR, 1.26; 95% CI, 1.02–1.55), in women this relationship was not observed (HR, 0.94; 95% CI, 0.61–1.35). In a cohort of 934 old adults over 65 years old from the In Chianti study it was discovered that the calf muscle area (as measured by peripheral quantitative CT) was not associated with a 6-years mortality (111).

In addition, also sarcopenic obesity was not associated with an increased mortality risk. Lastly, data from 3,153 65+ Chinese adults, showed similarities between sarcopenic and non-sarcopenic subjects in terms of 5-years mortality risk (144). On the whole, these studies systematically show that a higher mortality risk is not associated with a low muscle mass. Conversely, a recent study conducted during a 4.6-years follow-up found that the loss of appendicular muscle mass (as measured by DXA) was associated with an increased mortality risk in 4,331 males aged 65–93 years (116).

According to the available literature evidence, it is not possible to rule out the possibility that the increased mortality is actually caused by the weight loss experienced and the underlying cause of this loss, considering that in old adults the loss of skeletal muscle mass is strictly correlated with weight loss (154).

## Conclusions

The changes in BC occurring during lifetime are strictly related to health status. The increase of fat mass and the decrease of lean mass typical of elderly have indeed been associated with the increase of age-related pathologies and functional decline. A reduced mobility, the onset of disabilities and falls are among the major cause of reduced quality of life among elderly. Moreover, the specific increase of visceral fat in abdomen and of the ectopic fat storage in other organs and tissues and the decrease of skeletal muscle mass have been associated with an increased pro-inflammatory status and insulin resistance that can further increase the risk of pathologies including CVD and T2D. For these reasons, the study of composition and distribution of body masses it is becoming urgent because the inclusion of information regarding quantity and quality of fat, lean, and bone tissues could personalize preventive strategies for age-related pathologies.

Anthropometric measures such as BMI, waist circumference, waist to hip ratio, underwater weighing and more recently bioelectrical impedance are widely used to measure BC because of easy application, low costs and avoid radiation exposure.

However, the precision and accuracy of these methods is rather low and the level of distinction among different components of body mass and compartments is poor.

To date, the use of imaging techniques such as US, CT, MRI and DXA in clinical, but also in research is increasing due to an elevated precision and accuracy associated with a satisfactory level of discrimination among body masses.

**Figure 5:**
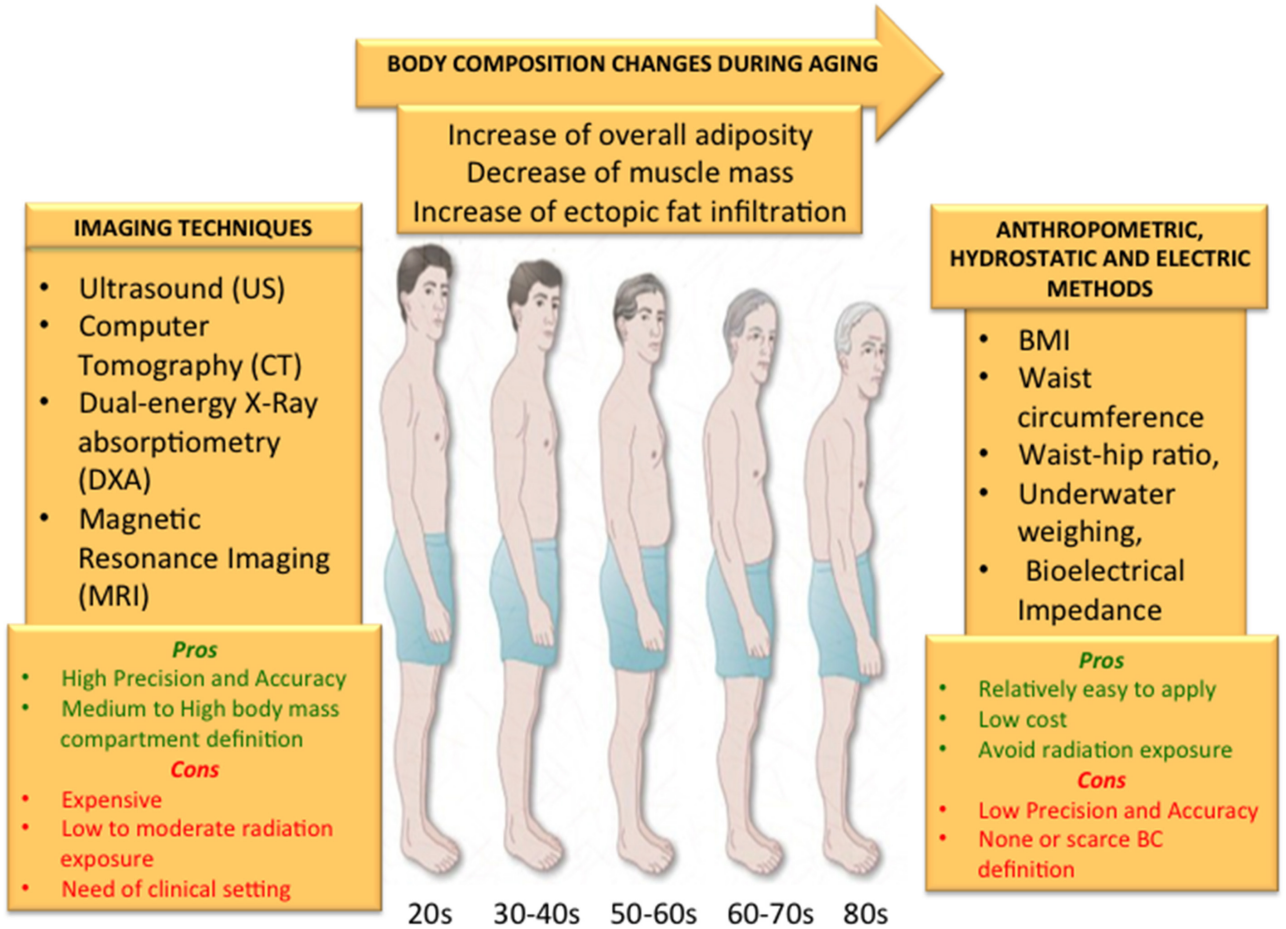
The changes that usually occur with age such as overall increase of body fat and ectopic fat infiltration and the decrease of skeletal muscle should be accurately measured in order to add this information to a personalized preventive strategy to counteract age-related disease and disabilities. Although anthropometric measures, underwater weighting and electric bioimpedance represent cheap, easy and completely safe methods, they do not guarantee a high precision and accuracy to define BC compartments with none or a scarce definition. On the other hand, imaging techniques can guarantee a very high definition of body compartments either in fat or lean mass with a high accuracy and precision. However, all the imaging methods expose the subjects to low or medium levels of radiation, are not easily available and are quite expensive. Depending on the information sought, all these aspects should be taken into account when selecting the method to measure BC.

However, depending on the information requested, specific advantages and limitations could be envisaged (Figure 5):

- MRI and CT are imaging modalities that provide very precise and accurate information for whole body, inter/intramuscular and visceral fat and for whole body and regional muscle but they both require a clinical setting, thus their availability is low, they are quite expensive and the exposure to radiation is high, in particular for CT;
- DXA provides images of whole-body fat and regional muscle with high precision and accuracy as well as MRI and CT. It is the most widely used technique for the study of bone composition and even if it requires a clinical setting it is relatively available and cheap, while involving a very low exposure to radiation;
- US is mainly used to measure abdominal adiposity. It is a low cost technique and avoids exposure to radiation, however its accuracy and reliability is still debated.

Collectively, imaging techniques are very promising in the study of BC and age-related changes. However, further efforts are needed to decrease the costs and thus increase the availability to population.

Lastly, the creation of standardized reference normative databases should be encouraged among researchers and to this aim a valid method for the cross-calibration among different scanners should be established to compare results among different research centers.

## Author Contributions

FP, AB, and AS contributed to the concept, analysis of literature, and writing of the manuscript. DM, CG, MC, and MM contributed to the analysis of literature. CF and LS contributed to the analysis of literature and critical discussion. All authors reviewed and/or edited the manuscript before submission.

### Conflict of Interest

The authors declare that the research was conducted in the absence of any commercial or financial relationships that could be construed as a potential conflict of interest.

## References

[1] World Health Organization World Report on Ageing and Health. World Health Organization (2015).

[2] Kennedy BK Berger SL Brunet A Campisi J Maria A Epel ES Aging: a common driver of chronic diseases and a target for novel interventions. Cell. (2014) 159:709–13. 10.1016/j.cell.2014.10.039 25417146 PMC4852871

[3] Franceschi C Garagnani P Parini P Giuliani C Santoro A. Inflammaging: a new immune–metabolic viewpoint for age-related diseases. Nat Rev Endocrinol. (2018) 14:576–90. 10.1038/s41574-018-0059-4 30046148

[4] Franceschi C Bonafè M Valensin S Olivieri F De Luca M Ottaviani E. Inflamm-aging. An evolutionary perspective on immunosenescence. Ann N Y Acad Sci. (2000) 908:244–54. 10.1111/j.1749-6632.2000.tb06651.x 10911963

[5] Franceschi C Zaikin A Gordleeva S Ivanchenko M Bonifazi F Storci G Seminars in immunology. Semin Immunol. (2018) 40:1–5. 10.1016/j.smim.2018.10.008 30392751

[6] Hotamisligil GS Inflammation and metabolic disorders. Nature. (2006) 14:860–7. 10.1038/nature05485 17167474

[7] Vitale G Franceschi C Salvioli S. Oxidative stress and the ageing endocrine system. Nat Rev Endocrinol. (2013) 9:228–40. 10.1038/nrendo.2013.29 23438835

[8] Franceschi C. Healthy ageing in 2016: obesity in geroscience — is cellular senescence the culprit? Nat Rev Endocrinol. (2017) 13:76–8. 10.1038/nrendo.2016.213 28059157

[9] Prattichizzo F De Nigris V Spiga R Mancuso E La Sala L Antonicelli R. Inflammageing and metaflammation: the yin and yang of type 2 diabetes. Ageing Res Rev. (2018) 41:1–17. 10.1016/j.arr.2017.10.003 29081381

[10] Hotamisligil GS. Inflammation, metaflammation and immunometabolic disorders. Nature. (2017) 542:177–85. 10.1038/nature21363 28179656

[11] Hotamisligil GS Shargill N Spiegelman BM dipose expression of tumor necrosis factor-alpha: direct role in obesity-linked insulin resistance. Science. (1993) 1:87–91. 10.1126/science.7678183 7678183

[12] Fried SK Bunkin DA Greenberg AS. Omental and subcutaneous adipose tissues of obese subjects release interleukin-6: depot difference and regulation by glucocorticoid. J Clin Endocrinol Metab. (1998) 83:847–50. 10.1210/jc.83.3.847 9506738

[13] Kanda H Tateya S Tamori Y Kotani K Hiasa K Kitazawa R. MCP-1 contributes to macrophage infiltration into adipose tissue, insulin resistance, and hepatic steatosis in obesity. J Clin Investig. (2006) 116:1494–505. 10.1172/JCI26498 16691291 PMC1459069

[14] Franceschi C Ostan R Santoro A. Nutrition and inflammation: are centenarians similar to individuals on calorie-restricted diets? Ann Rev Nutr. (2018) 38:329–56. 10.1146/annurev-nutr-082117-051637 29852087

[15] Zong G Zhang Z Yang Q Wu H Hu FB Sun Q. Total and regional adiposity measured by dual-energy X-ray absorptiometry and mortality in NHANES 1999-2006. HHS Public Access. (2017) 24:2414–21. 10.1002/oby.21659 27667735 PMC5117479

[16] Reinders I Visser M Schaap L. Body weight and body composition in old age and their relationship with frailty. Curr Opin Clin Nutr Metab Care. (2017) 20:11–5. 10.1097/MCO.0000000000000332 27749713

[17] Gavi S Stuart L Kelly P Melendez MM Mynarcik D Gelato M. Retinol-binding protein 4 is associated with insulin resistance and body fat distribution in nonobese subjects without type 2 diabetes. J Clin Endocrinol Metab. (2007) 92:1886–90. 10.1210/jc.2006-1815 17299074

[18] Conte M Martucci M Sandri M Franceschi C Salvioli S. The dual role of the pervasive “Fattish” tissue remodeling with age. Front Endocrinol. (2019) 10:114. 10.3389/fendo.2019.00114 30863366 PMC6400104

[19] Santoro A Bazzocchi A Guidarelli G Ostan R Giampieri E Mercatelli D. A cross-sectional analysis of body composition among healthy elderly from the European NU-AGE study: sex and country specific features. Front Physiol. (2018) 9:1963. 10.3389/fphys.2018.01693 30555339 PMC6283977

[20] Ross R. Advances in the application of imaging methods in applied and clinical physiology. Acta Diabetol. (2003) 40:s45–50. 10.1007/s00592-003-0025-y 14618432

[21] Thomas EL Saeed N Hajnal JV Brynes A Goldstone AP Frost G. Magnetic resonance imaging of total body fat. J Appl Physiol. (1998) 85:1778–85. 10.1152/jappl.1998.85.5.1778 9804581

[22] Bazzocchi A Diano D Ponti F Andreone A Sassi C Albisinni U. Health and ageing: a cross-sectional study of body composition. Clin Nutr. (2013) 32:569–78. 10.1016/j.clnu.2012.10.004 23111003

[23] Guglielmi G Ponti F Agostini M Amadori M Battista G Bazzocchi A. The role of DXA in sarcopenia. Aging Clin Exp Res. (2016) 28:1047–60. 10.1007/s40520-016-0589-3 27256078

[24] Hangartner TN Warner S Braillon P Jankowski L Shepherd J. The official positions of the international society for clinical densitometry: acquisition of dual-energy X-ray absorptiometry body composition and considerations regarding analysis and repeatability of measures. J Clin Densitometry. (2013) 16:520–36. 10.1016/j.jocd.2013.08.007 24183641

[25] Bazzocchi A Ponti F Albisinni U Battista G Guglielmi G. DXA: technical aspects and application. Eur J Radiol. (2016) 85:1481–92. 10.1016/j.ejrad.2016.04.004 27157852

[26] Lewiecki EM Binkley N Morgan SL Shuhart CR Camargos BM Carey JJ. Best practices best practices for dual-energy X-ray absorptiometry measurement and reporting: international society for clinical densitometry guidance. J Clin Densitometry. (2016) 19:127–40. 10.1016/j.jocd.2016.03.003 27020004

[27] Guerri S Mercatelli D Aparisi Gómez MP Napoli A Battista G Guglielmi G. Quantitative imaging techniques for the assessment of osteoporosis and sarcopenia. Quant Imaging Med Surg. (2018) 8:60–85. 10.21037/qims.2018.01.05 29541624 PMC5835658

[28] Santoro A Pini E Scurti M Palmas G Berendsen A Brzozowska A. Combating inflammaging through a Mediterranean whole diet approach: the NU-AGE project's conceptual framework and design. Mech Ageing Dev. (2014) 136–137:3–13. 10.1016/j.mad.2013.12.001 24342354

[29] Santoro A Guidarelli G Ostan R Giampieri E Fabbri C Bertarelli C. Gender-specific association of body composition with inflammatory and adipose-related markers in healthy elderly Europeans from the NU-AGE study. Eur Radiol. (2019) 29:4968–79. 10.1007/s00330-018-5973-2 30715588 PMC6682581

[30] Kelly TL Wilson KE Heymsfield SB. Dual energy X-ray absorptiometry body composition reference values from NHANES. PLoS ONE. (2009) 4:e7038. 10.1371/journal.pone.0007038 19753111 PMC2737140

[31] Bazzocchi A Filonzi G Ponti F Albisinni U Guglielmi G Battista G. Ultrasound: Which role in body composition? Eur J Radiol. (2016) 85:1469–80. 10.1016/j.ejrad.2016.04.005 27235340

[32] Bazzocchi A Filonzi G Ponti F Sassi C Salizzoni E Battista G. Accuracy, reproducibility and repeatability of ultrasonography in the assessment of abdominal adiposity. Acad Radiol. (2011) 18:1133–43. 10.1016/j.acra.2011.04.014 21724427

[33] Borkan GA Hults DE Cardarelli J Burrows BA. Comparison of ultrasound and skinfold measurements in assessment of subcutaneous and total fatness. Am J Phys Anthropol. (1982) 58:307–13. 10.1002/ajpa.1330580309 7124924

[34] Fanelli MT Kuczmarski RJ. Ultrasound as an approach to assessing body composition. Am J Clin Nutr. (1984) 39:703–9. 10.1093/ajcn/39.5.703 6711473

[35] Armellini F Zamboni M Rigo L Bergamo-Andreis IA Robbi R De Marchi M. Sonography detection of small intra-abdominal fat variations. Int J Obesity. (1991) 15:847–52. 1794927

[36] Armellini F Zamboni M Robbi R Todesco T Rigo L Bergamo-Andreis IA. Total and intra-abdominal fat measurements by ultrasound and computerized tomography. Int J Obes Relat Metab Disord. (1993) 17:209–14 8387970

[37] Bazzocchi A Filonzi G Ponti F Amadori M Sassi C Salizzoni E. The role of ultrasonography in the evaluation of abdominal fat: analysis of technical and methodological issues. Acad Radiol. (2013) 20:1278–85. 10.1016/j.acra.2013.07.009 24029060

[38] Malietzis G Aziz O Bagnall NM Johns N Fearon KC Jenkins JT. The role of body composition evaluation by computerized tomography in determining colorectal cancer treatment outcomes: a systematic review. Eur J Surg Oncol. (2015) 41:186–96. 10.1016/j.ejso.2014.10.056 25468746

[39] Eastwood SV Tillin T Wright A Heasman J Willis J Godsland IF. Estimation of CT-derived abdominal visceral and subcutaneous adipose tissue depots from anthropometry in Europeans, South Asians and African Caribbeans. PLoS ONE. (2013) 8:e75085. 10.1371/journal.pone.0075085 24069381 PMC3775834

[40] Wang H Chen YE Eitzman DT. Imaging body fat: techniques and cardiometabolic implications. Arteriosclerosis Thrombosis Vascu Biol. (2014) 34:2217–23. 10.1161/ATVBAHA.114.303036 25147343 PMC4169325

[41] Lemos T Gallagher D. Current body composition measurement techniques. Curr Opin Endocrinol Diabet Obes. (2017) 24:310–4. 10.1097/MED.0000000000000360 28696961 PMC5771660

[42] Hu HH Kan HE. Quantitative proton MR techniques for measuring fat. NMR Biomed. (2013) 26:1609–29. 10.1002/nbm.3025 24123229 PMC4001818

[43] Seabolt LA Welch EB Silver HJ. Imaging methods for analyzing body composition in human obesity and cardiometabolic disease. Ann N Y Acad Sci. (2015) 1353:41–59. 10.1111/nyas.12842 26250623

[44] Thomas EL Fitzpatrick JA Malik SJ Taylor-Robinson SD Bell JD. Whole body fat: content and distribution. Prog Nuclear Magnet Reson Spectrosc. (2013) 73:56–80. 10.1016/j.pnmrs.2013.04.001 23962884

[45] Borga M. MRI adipose tissue and muscle composition analysis-a review of automation techniques. Br J Radiol. (2018) 91:20180252. 10.1259/bjr.20180252 30004791 PMC6223175

[46] Aparisi Gómez MP Ponti F Mercatelli D Gasperini C Napoli A Battista G Correlation between DXA and laboratory parameters in normal weight, overweight, and obese patients. Nutrition. (2019) 61:143–50. 10.1016/j.nut.2018.10.023 30711863

[47] Shen W Chen J Gantz M Velasquez G Punyanitya M Heymsfield SB A single MRI slice does not accurately predict visceral and subcutaneous adipose tissue changes during weight loss. Obesity. (2012) 20:2458–63. 10.1038/oby.2012.168 22728693 PMC3466347

[48] Yang YX Chong MS Lim WS Tay L Yew S Yeo A. Validity of estimating muscle and fat volume from a single MRI section in older adults with sarcopenia and sarcopenic obesity. Clin Radiol. (2017) 72:427.e9–427.e14. 10.1016/j.crad.2016.12.011 28117037

[49] Gloor M Fasler S Fischmann A Haas T Bieri O Heinimann K. Quantification of fat infiltration in oculopharyngeal muscular dystrophy: comparison of three MR imaging methods. J Magnet Reson Imaging. (2011) 33:203–10. 10.1002/jmri.22431 21182140

[50] Napolitano A Miller SR Murgatroyd PR Coward WA Wright A Finer N. Validation of a quantitative magnetic resonance method for measuring human body composition. Obesity. (2008) 16:191–8. 10.1038/oby.2007.29 18223634

[51] Addison O Drummond MJ LaStayo PC Dibble LE Wende AR McClain DA. Intramuscular fat and inflammation differ in older adults: the impact of frailty and inactivity. J Nutr Health Aging. (2014) 18:532–8. 10.1007/s12603-014-0019-1 24886741 PMC6639009

[52] Wong AKO Beattie KA Min KKH Gordon C Pickard L Papaioannou A. Peripheral quantitative computed tomography-derived muscle density and peripheral magnetic resonance imaging-derived muscle adiposity: precision and associations with fragility fractures in women. J Muscul Neuronal Inter. (2014) 14:401–10. 25524965 PMC5092150

[53] Lorbergs AL Noseworthy MD Adachi JD Stratford PW MacIntyre NJ. Fat infiltration in the leg is associated with bone geometry and physical function in healthy older women. Calcified Tissue Int. (2015) 97:353–63. 10.1007/s00223-015-0018-1 26071112

[54] Cruz-Jentoft AJ Baeyens JP Bauer JM Boirie Y Cederholm T Landi F. Sarcopenia: European consensus on definition and diagnosis Report of the European Working Group on Sarcopenia in older people. Age Ageing. (2010) 39:412–23. 10.1093/ageing/afq034 20392703 PMC2886201

[55] Erlandson MC Lorbergs AL Mathur S Cheung AM. Muscle analysis using pQCT, DXA and MRI. Eur J Radiol. (2016) 85:1505–11. 10.1016/j.ejrad.2016.03.001 27005009

[56] Drøyvold WB Nilsen TIL Krüger O Holmen TL Krokstad S Midthjell K. Change in height, weight and body mass index: longitudinal data from the HUNT Study in Norway. Int J Obes. (2006) 30:935–9. 10.1038/sj.ijo.0803178 16418765

[57] Visser M Deeg DJH The effect of age-related height loss on the BMI classification of older men and women. Int J Body Compos Res. (2007) 5:35–40.

[58] Lee JS Kritchevsky SB Tylavsky F Harris T Simonsick EM Rubin SM. Weight change, weight change intention, and the incidence of mobility limitation in well-functioning community-dwelling older adults. J Gerontol A Biol Sci Med Sci. (2005) 60:1007–12. 10.1093/gerona/60.8.1007 16127104

[59] Arnold AM Newman AB Cushman M Ding J Kritchevsky S Body weight dynamics and their association with physical function and mortality in older adults: the cardiovascular health study. J Gerontol A Biol Sci Med Sci. (2010) 65A:63–70. 10.1093/gerona/glp050 PMC2796878 19386574

[60] Ding J Kritchevsky SB Newman AB Taaffe DR Nicklas BJ Visser M Effects of birth cohort and age on body composition in a sample of community-based elderly 1-3. Am J Clin Nutr. (2007) 85:405–10. 10.1093/ajcn/85.2.405 17284736

[61] Gallagher D Ruts E Visser M Heshka S Baumgartner RN Wang J. Weight stability masks sarcopenia in elderly men and women. Am J Physiol Endocrinol Metab. (2000) 279:E366–75. 10.1152/ajpendo.2000.279.2.E366 10913037

[62] Zamboni M Zoico E Scartezzini T Mazzali G Tosoni P Zivelonghi A. Body composition changes in stable-weight elderly subjects: the effect of sex. Aging Clin Exp Res. (2003) 15:321–7. 10.1007/BF03324517 14661824

[63] Koster A Ding J Stenholm S Caserotti P Houston DK Nicklas BJ. Does the amount of fat mass predict age-related loss of lean mass, muscle strength, and muscle quality in older adults? J Gerontol Series A Biol Sci Med Sci. (2011) 66:888–95. 10.1093/gerona/glr070 21572082 PMC3184893

[64] Newman AB Lee JS Visser M Goodpaster BH Kritchevsky SB Tylavsky FA. Weight change and the conservation of lean mass in old age: the health, aging and body composition study. Am J Clin Nutr. (2005) 82:872–8. 10.1093/ajcn/82.4.872 16210719

[65] Bazzocchi A Ponti F Diano D Moio A Albisinni U Pasquali R. Abdominal adiposity by ultrasonography: a “pocket” database for reference standard in Italian people. Primary Care Diabetes. (2014) 8:358–64. 10.1016/j.pcd.2014.02.003 24636921

[66] Song M-Y Ruts E Kim J Janumala I Heymsfield S Gallagher D. Sarcopenia and increased adipose tissue infiltration of muscle in elderly African American women. Am J Clin Nutr. (2004) 79:874–80. 10.1093/ajcn/79.5.874 15113728

[67] Delmonico MJ Harris TB Visser M Park SW Conroy MB Velasquez-Mieyer P. Longitudinal study of muscle strength, quality, and adipose tissue infiltration. Am J Clin Nutr. (2009) 90:1579–85. 10.3945/ajcn.2009.28047 19864405 PMC2777469

[68] Rossi AP Watson NL Newman AB Harris TB Kritchevsky SB Bauer DC. Effects of body composition and adipose tissue distribution on respiratory function in elderly men and women: the health, aging, and body composition study. J Gerontol A Biol Sci Med Sci. (2011) 66:801–8. 10.1093/gerona/glr059 21498841 PMC3143349

[69] Forbes GB Reina JC. Adult lean body mass declines with age: Some longitudinal observations. Metabolism. (1970) 19:653–63. 10.1016/0026-0495(70)90062-4 5459997

[70] Rosenberg IH. Sarcopenia: origins and clinical relevance. J Nutr. (1997) 127:990S−1S. 10.1093/jn/127.5.990S 9164280

[71] VisserM. Towards a definition of sarcopenia–results from epidemiologic studies. J Nutr Health Aging. (2009) 13:713–6. 10.1007/s12603-009-0202-y19657555

[72] Frontera WR Reid KF Phillips EM Krivickas LS Hughes VA Roubenoff R. Muscle fiber size and function in elderly humans: a longitudinal study. J Appl Physiol. (2008) 105:637–42. 10.1152/japplphysiol.90332.2008 18556434 PMC2519941

[73] Hughes VA Frontera WR Wood M Evans WJ Dallal GE Roubenoff R. Longitudinal muscle strength changes in older adults: influence of muscle mass, physical activity, and health. J Gerontol Series A Biol Sci Med Sci. (2001) 56:B209–17. 10.1093/gerona/56.5.B209 11320101

[74] Fantin F Di Francesco V Fontana G Zivelonghi A Bissoli L Zoico E. Longitudinal body composition changes in old men and women: interrelationships with worsening disability. J Gerontol Series A Biol Sci Med Sci. (2007) 62:1375–81. 10.1093/gerona/62.12.1375 18166688

[75] Zamboni M Mazzali G Fantin F Rossi A Di Francesco V. Sarcopenic obesity: a new category of obesity in the elderly. Nutr Metab Cardiovasc Dis. (2008) 18:388–95. 10.1016/j.numecd.2007.10.002 18395429

[76] Stenholm S Harris TB Rantanen T Visser M Kritchevsky SB Ferrucci L. Sarcopenic obesity: definition, cause and consequences. Curr Opin Clin Nutr MetabCare. (2008) 11:693–700. 10.1097/MCO.0b013e328312c37d 18827572 PMC2633408

[77] Diano D Ponti F Guerri S Mercatelli D Amadori M Aparisi Gómez MP. Upper and lower limbs composition: a comparison between anthropometry and dual-energy X-ray absorptiometry in healthy people. Arch Osteoporosis. (2017) 12:78. 10.1007/s11657-017-0374-8 28921453

[78] Koster A Patel KV Visser M Van Eijk JTM Kanaya AM De Rekeneire N. Joint effects of adiposity and physical activity on incident mobility limitation in older adults. J Am Geriatr Soc. (2008) 56:636–43. 10.1111/j.1532-5415.2007.01632.x 18284534

[79] Vincent HK Vincent KR Lamb KM. Obesity and mobility disability in the older adult. Obes Rev. (2010) 11:568–79. 10.1111/j.1467-789X.2009.00703.x 20059707

[80] Jensen GL Hsiao PY. Obesity in older adults: relationship to functional limitation. Curr Opin Clin Nutr Metab Care. (2010) 13:46–51. 10.1097/MCO.0b013e32833309cf 19841579

[81] Marsh AP Rejeski WJ Espeland MA Miller ME Church TS Fielding RA. Muscle strength and BMI as predictors of major mobility disability in the Lifestyle Interventions and Independence for Elders pilot (LIFE-P). J Gerontol Series A Biol Sci Med Sci. (2011) 66:1376–83. 10.1093/gerona/glr158 21975090 PMC3210962

[82] Visser M Langlois J Guralnik JM Cauley JA Kronmal RA Robbins J High body fatness, but not low fat-free mass, predicts disability in older men and women: the Cardiovascular Health Study. Am J Clin Nutr. (1998) 68:584–90. 10.1093/ajcn/68.3.584 9734734

[83] Broadwin J Goodman-Gruen D Slymen D. Ability of fat and fat-free mass percentages to predict functional disability in older men and women. J Am Geriatr Soc. (2001) 49:1641–5. 10.1111/j.1532-5415.2001.49273.x 11843997

[84] Visser M Goodpaster BH Kritchevsky SB Newman AB Nevitt M Rubin SM. Muscle mass, muscle strength, and muscle fat infiltration as predictors of incident mobility limitations in well-functioning older persons. J Gerontol Series A Biol Sci Med Sci. (2005) 60:324–33. 10.1093/gerona/60.3.324 15860469

[85] Zoico E Di Francesco V Mazzali G Zivelonghi A Volpato S Bortolani A. High baseline values of fat mass, independently of appendicular skeletal mass, predict 2-year onset of disability in elderly subjects at the high end of the functional spectrum. Aging Clin Exp Res. (2007) 19:154–9. 10.1007/BF03324682 17446727

[86] Houston DK Ding J Nicklas BJ Harris TB Lee JS Nevitt MC. Overweight and obesity over the adult life course and incident mobility limitation in older adults: the health, aging and body composition study. Am J Epidemiol. (2009) 169:927–36. 10.1093/aje/kwp007 19270048 PMC2727232

[87] Stenholm S Rantanen T Alanen E Reunanen A Sainio P Koskinen S. Obesity history as a predictor of walking limitation at old age. Obesity. (2007) 15:929–38. 10.1038/oby.2007.583 17426328

[88] Fine JT Colditz GA Coakley EH Moseley G Manson JE Willett WC. A prospective study of weight change and health-related quality of life in women. JAMA. (1999) 282:2136–42. 10.1001/jama.282.22.2136 10591335

[89] Busetto L Romanato G Zambon S Calò E Zanoni S Corti MC. The effects of weight changes after middle age on the rate of disability in an elderly population sample. J Am Geriatr Soc. (2009) 57:1015–21. 10.1111/j.1532-5415.2009.02273.x 19507294

[90] Launer LJ Harris T Rumpel C Madan J. Body mass index, weight change, and risk of mobility disability in middle-aged and older women. The epidemiologic follow-up study of NHANES I. JAMA. (1994) 271:1093–8. 10.1001/jama.271.14.1093 8151851

[91] Lee JS Kritchevsky SB Harris TB Tylavsky F Rubin SM Newman AB. Short-term weight changes in community-dwelling older adults: the Health, Aging, and Body Composition Weight Change Substudy. Am J Clin Nutr. (2005) 82:644–50. 10.1093/ajcn/82.3.644 16155279

[92] Villareal DT Chode S Parimi N Sinacore DR Hilton T Armamento-Villareal R Weight loss, exercise, or both and physical function in obese older adults. N Engl J Med. (2011) 364:1218–47. 10.1056/NEJMoa1008234 21449785 PMC3114602

[93] Villareal DT Apovian CM Kushner RF Klein S American Society for Nutrition, NAASO. Obesity in older adults: technical review and position statement of the american society for nutrition and NAASO, the obesity society. Obes Res. (2005) 13:1849–63. 10.1038/oby.2005.228 16339115

[94] Flegal KM Kit BK Orpana H Graubard BI. Association of all-cause mortality with overweight and obesity using standard body mass index categories: a systematic review and meta-analysis. JAMA. (2013) 309:71–82. 10.1001/jama.2012.113905 23280227 PMC4855514

[95] DavidCNde MelloRBBruscatoNMMoriguchiEH. Overweight and abdominal obesity association with all-cause and cardiovascular mortality in the elderly aged 80 and over: a cohort study. J Nutr Health Aging. (2017) 21:597–603. 10.1007/s12603-016-0812-028448093

[96] Klatsky AL Zhang J Udaltsova N Li Y Tran HN. Body mass index and mortality in a very large cohort: is it really healthier to be overweight? Permanente J. (2017) 21:16–142. 10.7812/TPP/16-142 28678695 PMC5499607

[97] Kalish VB. Obesity in older adults. Primary Care Clin Office Pract. (2016) 43:137–44. 10.1016/j.pop.2015.10.002 26896206

[98] Bhaskaran K dos-Santos-Silva I Leon DA Douglas IJ Smeeth L. Association of BMI with overall and cause-specific mortality: a population-based cohort study of 3·6 million adults in the UK. Lancet Diabetes Endocrinol. (2018) 6:944–53. 10.1016/S2213-8587(18)30288-2 30389323 PMC6249991

[99] Janssen I Mark AE. Elevated body mass index and mortality risk in the elderly. Obes Rev. (2007) 8:41–59. 10.1111/j.1467-789X.2006.00248.x 17212795

[100] Reis JP Macera CA Araneta MR Lindsay SP Marshall SJ Wingard DL. Comparison of overall obesity and body fat distribution in predicting risk of mortality. Obesity. (2009) 17:1232–9. 10.1038/oby.2008.664 19197258

[101] Adams KF Schatzkin A Harris TB Kipnis V Mouw T Ballard-Barbash R. Overweight, obesity, and mortality in a large prospective cohort of persons 50 to 71 years old. N Engl J Med. (2006) 355:763–78. 10.1056/NEJMoa055643 16926275

[102] Sui X LaMonte MJ Laditka JN Hardin JW Chase N Hooker SP. Cardiorespiratory fitness and adiposity as mortality predictors in older adults. JAMA. (2007) 298:2507–16. 10.1001/jama.298.21.2507 18056904 PMC2692959

[103] Singh PN Haddad E Tonstad S Fraser GE. Does excess body fat maintained after the seventh decade decrease life expectancy? J Am Geriatr Soc. (2011) 59:1003–11. 10.1111/j.1532-5415.2011.03419.x 21649624

[104] Shaukat A Dostal A Menk J Church TR. BMI is a risk factor for colorectal cancer mortality. Digest Dis Sci. (2017) 62:2511–7. 10.1007/s10620-017-4682-z 28733869

[105] Leigh L Byles JE Jagger C. BMI and healthy life expectancy in old and very old women. Br J Nutr. (2016) 116:692–9. 10.1017/S0007114516002403 27307012

[106] Ng TP Jin A Chow KY Feng L Nyunt MSZ Yap KB. Age-dependent relationships between body mass index and mortality: Singapore longitudinal ageing study. PLoS One. (2017) 12:e0180818. 10.1371/journal.pone.0180818 28738068 PMC5524359

[107] Thinggaard M Jacobsen R Jeune B Martinussen T Christensen K. Is the relationship between BMI and mortality increasingly U-shaped with advancing age? A 10-year follow-up of persons aged 70-95 years. J Gerontol Series A Biol Sci Med Sci. (2010) 65:526–31. 10.1093/gerona/glp214 20089666 PMC2854881

[108] Lisko I Tiainen K Stenholm S Luukkaala T Hervonen A Jylha M Body mass index, waist circumference, and waist-to-hip ratio as predictors of mortality in nonagenarians: the vitality 90+ study. J Gerontol Series A Biol Sci Med Sci. (2011) 66A:1244–50. 10.1093/gerona/glr147 21860016

[109] Heitmann BL Erikson H Ellsinger BM Mikkelsen KL Larsson B. Mortality associated with body fat, fat-free mass and body mass index among 60-year-old swedish men-a 22-year follow-up. The study of men born in 1913. Int J Obes Relat Metab Disord. (2000) 24:33–7. 10.1038/sj.ijo.0801082 10702748

[110] Bigaard J Frederiksen K Tjønneland A Thomsen BL Overvad K Heitmann BL. Waist circumference and body composition in relation to all-cause mortality in middle-aged men and women. Int J Obes. (2005) 29:778–84. 10.1038/sj.ijo.0802976 15917857

[111] Cesari M Pahor M Lauretani F Zamboni V Bandinelli S Bernabei R. Skeletal muscle and mortality results from the InCHIANTI Study. J Gerontol Series A Biol Sci Med Sci. (2009) 64:377–84. 10.1093/gerona/gln031 19181709 PMC2655006

[112] Auyeung TW Lee JSW Leung J Kwok T Leung PC Woo J. Survival in older men may benefit from being slightly overweight and centrally obese–a 5-year follow-up study in 4,000 older adults using DXA. J Gerontol Series A Biol Sci Med Sci. (2010) 65:99–104. 10.1093/gerona/glp099 19628635 PMC2796879

[113] Reynolds MW Fredman L Langenberg P Magaziner J. Weight, weight change, and mortality in a random sample of older community-dwelling women. J Am Geriatr Soc. (1999) 47:1409–14. 10.1111/j.1532-5415.1999.tb01558.x 10591233

[114] Newman AB Yanez D Harris T Duxbury A Enright PL Fried LP. Weight change in old age and its association with mortality. J Am Geriatr Soc. (2001) 49:1309–18. 10.1046/j.1532-5415.2001.49258.x 11890489

[115] Amador LF Al Snih S Markides KS Goodwin JS. Weight change and mortality among older Mexican Americans. Aging Clin Exp Res. (2006) 18:196–204. 10.1007/BF03324649 16804365

[116] Lee CG Boyko EJ Nielson CM Stefanick ML Bauer DC Hoffman AR. Mortality risk in older men associated with changes in weight, lean mass, and fat mass. J Am Geriatr Soc. (2011) 59:233–40. 10.1111/j.1532-5415.2010.03245.x 21288234 PMC3403719

[117] Shea MK Houston DK Nicklas BJ Messier SP Davis CC Miller ME. The effect of randomization to weight loss on total mortality in older overweight and obese adults: the ADAPT Study. J Gerontol Series A Biol Sci Med Sci. (2010) 65:519–25. 10.1093/gerona/glp217 20080875 PMC3107029

[118] Park S-Y Wilkens LR Maskarinec G Haiman CA Kolonel LN Marchand LL. Weight change in older adults and mortality: the Multiethnic Cohort Study. Int J Obes. (2018) 42:205–12. 10.1038/ijo.2017.188 28885999 PMC5803382

[119] Okoro CA Zhong Y Ford ES Balluz LS Strine TW Mokdad AH. Association between the metabolic syndrome and its components and gait speed among U.S. adults aged 50 years and older: a cross-sectional analysis. BMC public health. (2006) 6:282. 10.1186/1471-2458-6-282 17105659 PMC1654157

[120] Ramsay SE Whincup PH Shaper AG Wannamethee SG. The relations of body composition and adiposity measures to ill health and physical disability in elderly men. Am J Epidemiol. (2006) 164:459–69. 10.1093/aje/kwj217 16818465

[121] Chen H Guo X. Obesity and functional disability in elderly Americans. J Am Geriatr Soc. (2008) 56:689–94. 10.1111/j.1532-5415.2007.01624.x 18266843 PMC2391089

[122] Bouchard DR Choquette S Dionne IJ Brochu M. Is fat mass distribution related to impaired mobility in older men and women? Nutrition as a determinant of successful aging: the Quebec longitudinal study. Exp Aging Res. (2011) 37:346–57. 10.1080/0361073X.2011.568848 21534033

[123] Meadows R Bower JK. Associations of anthropometric measures of obesity with physical limitations in older adults. Disab Rehabil. (2018). 10.1080/09638288.2018.1516815. [Epub ahead of print]. 30574808

[124] Bannerman E Miller MD Daniels LA Cobiac L Giles LC Whitehead C. Anthropometric indices predict physical function and mobility in older Australians: the Australian Longitudinal Study of Ageing. Public Health Nutr. (2002) 5:655–62. 10.1079/PHN2002336 12372159

[125] Houston DK Stevens J Cai J. Abdominal fat distribution and functional limitations and disability in a biracial cohort: the Atherosclerosis Risk in Communities Study. Int J Obes. (2005) 29:1457–63. 10.1038/sj.ijo.0803043 16077713

[126] Guallar-Castillón P Sagardui-Villamor J Banegas JR Graciani A Fornés NS López García E. Waist Circumference as a Predictor of Disability among Older Adults^*^. Obesity. (2007) 15:233. 10.1038/oby.2007.532 17228052

[127] Angleman SB Harris TB Melzer D. The role of waist circumference in predicting disability in periretirement age adults. Int J Obes. (2006) 30:364–73. 10.1038/sj.ijo.0803130 16231023

[128] Corona LP Alexandre TS Duarte YAO Lebrão ML. Abdominal obesity as a risk factor for disability in Brazilian older adults. Public Health Nutr. (2017) 20:1046–53. 10.1017/S1368980016003505 28112078 PMC10261643

[129] Yang M Jiang J Li H Wu H Dong B. Association between waist circumference and self-reported disability among Chinese adults aged 90 years and older. Geriatr Gerontol Int. (2015) 15:1249–57. 10.1111/ggi.12424 25496442

[130] Fernandes de Souza BarbosaJDos Santos GomesCVilton CostaJAhmedTZunzuneguiMVCurcioC-L. Abdominal obesity and mobility disability in older adults: a 4-year follow-up the international mobility in aging study. J Nutr Health Aging. (2018) 22:1228–37. 10.1007/s12603-018-1100-y30498831

[131] Goodpaster BH Carlson CL Visser M Kelley DE Scherzinger A Harris TB. Attenuation of skeletal muscle and strength in the elderly: The Health ABC Study. J Appl Physiol. (2001) 90:2157–65. 10.1152/jappl.2001.90.6.2157 11356778

[132] Reinders I Murphy RA Koster A Brouwer IA Visser M Garcia ME. Muscle quality and muscle fat infiltration in relation to incident mobility disability and gait speed decline: the age, gene/environment Susceptibility-Reykjavik Study. J Gerontol Series A Biol Sci Med Sci. (2015) 70:1030–6. 10.1093/gerona/glv016 25748031 PMC4506318

[133] Scott D Trbojevic T Skinner E Clark RA Levinger P Haines TP. Associations of calf inter- and intra-muscular adipose tissue with cardiometabolic health and physical function in community-dwelling older adults. J Musculoskeletal Neuronal Interact. (2015) 15:350–7. 26636281 PMC5628595

[134] Wannamethee SG Shaper AG Lennon L Whincup PH. Decreased muscle mass and increased central adiposity are independently related to mortality in older men. Am J Clin Nutr. (2007) 86:1339–46. 10.1093/ajcn/86.5.1339 17991644

[135] Koster A Leitzmann MF Schatzkin A Mouw T Adams KF van Eijk JTM. Waist circumference and mortality. Am J Epidemiol. (2008) 167:1465–75. 10.1093/aje/kwn079 18417494

[136] Leitzmann MF Moore SC Koster A Harris TB Park Y Hollenbeck A. Waist circumference as compared with body-mass index in predicting mortality from specific causes. PLoS ONE. (2011) 6:e18582. 10.1371/journal.pone.0018582 21541313 PMC3082527

[137] Visscher TL Seidell JC Molarius A van der Kuip D Hofman A Witteman JC. A comparison of body mass index, waist-hip ratio and waist circumference as predictors of all-cause mortality among the elderly: the Rotterdam study. Int J Obes Relat Metab Disord. (2001) 25:1730–5. 10.1038/sj.ijo.0801787 11753597

[138] Pujilestari CU Nyström L Norberg M Ng N. Waist Circumference and All-cause mortality among older adults in rural Indonesia. Int J Environ Res Public Health. (2019) 16:116. 10.3390/ijerph16010116 30609857 PMC6339011

[139] Kuk JL Katzmarzyk PT Nichaman MZ Church TS Blair SN Ross R. Visceral fat is an independent predictor of all-cause mortality in men. Obesity. (2006) 14:336–41. 10.1038/oby.2006.43 16571861

[140] Baumgartner RN Koehler KM Gallagher D Romero L Heymsfield SB Ross RR. Epidemiology of Sarcopenia among the Elderly in New Mexico. Am J Epidemiol. (1998) 147:755–63. 10.1093/oxfordjournals.aje.a009520 9554417

[141] Janssen I Baumgartner RN Ross R Rosenberg IH Roubenoff R. Skeletal muscle cutpoints associated with elevated physical disability risk in older men and women. Am J Epidemiol. (2004) 159:413–21. 10.1093/aje/kwh058 14769646

[142] Newman AB Kupelian V Visser M Simonsick E Goodpaster B Nevitt M. Sarcopenia: alternative definitions and associations with lower extremity function. J Am Geriatr Soc. (2003) 51:1602–9. 10.1046/j.1532-5415.2003.51534.x 14687390

[143] Delmonico MJ Harris TB Lee J-S Visser M Nevitt M Kritchevsky SB. Alternative definitions of sarcopenia, lower extremity performance, and functional impairment with aging in older men and women. J Am Geriatr Soc. (2007) 55:769–74. 10.1111/j.1532-5415.2007.01140.x 17493199

[144] Woo J Leung J Sham A Kwok T. Defining sarcopenia in terms of risk of physical limitations: a 5-year follow-up study of 3,153 chinese men and women. J Am Geriatr Soc. (2009) 57:2224–31. 10.1111/j.1532-5415.2009.02566.x 19925615

[145] Visser M Harris TB Langlois J Hannan MT Roubenoff R Felson DT Body fat and skeletal muscle mass in relation to physical disability in very old men and women of the Framingham Heart Study. J Gerontol Series A Biol Sci Med Sci. (1998) 53A:M214–21. 10.1093/gerona/53A.3.M214 9597054

[146] Lebrun CEI van der Schouw YT de Jong FH Grobbee DE Lamberts SW. Fat mass rather than muscle strength is the major determinant of physical function and disability in postmenopausal women younger than 75 years of age. Menopause. (2006) 13:474–81. 10.1097/01.gme.0000222331.23478.ec 16735945

[147] Jankowski CM Gozansky WS Van Pelt RE Schenkman ML Wolfe P Schwartz RS. Relative contributions of adiposity and muscularity to physical function in community-dwelling older adults. Obesity. (2008) 16:1039–44. 10.1038/oby.2007.84 18292753 PMC4391797

[148] Rolland Y Lauwers-Cances V Cristini C Abellan van Kan G Janssen I Morley JE. Difficulties with physical function associated with obesity, sarcopenia, and sarcopenic-obesity in community-dwelling elderly women: the EPIDOS (EPIDemiologie de l'OSteoporose) Study. Am J Clin Nutr. (2009) 89:1895–900. 10.3945/ajcn.2008.26950 19369381

[149] Hairi NN Cumming RG Naganathan V Handelsman DJ Le Couteur DG Creasey H. Loss of muscle strength, mass (sarcopenia), and quality (specific force) and its relationship with functional limitation and physical disability: the Concord Health and Ageing in Men Project. J Am Geriatr Soc. (2010) 58:2055–62. 10.1111/j.1532-5415.2010.03145.x 21054284

[150] Baumgartner RN Wayne SJ Waters DL Janssen I Gallagher D Morley JE. Sarcopenic obesity predicts instrumental activities of daily living disability in the elderly. Obes Res. (2004) 12:1995–2004. 10.1038/oby.2004.250 15687401

[151] Davison KK Ford ES Cogswell ME Dietz WH. Percentage of body fat and body mass index are associated with mobility limitations in people aged 70 and older from NHANES III. J Am Geriatr Soc. (2002) 50:1802–9. 10.1046/j.1532-5415.2002.50508.x 12410898

[152] Zoico E Di Francesco V Guralnik JM Mazzali G Bortolani A Guariento S. Physical disability and muscular strength in relation to obesity and different body composition indexes in a sample of healthy elderly women. Int J Obes Relat Metab Disord. (2004) 28:234–41. 10.1038/sj.ijo.0802552 14708033

[153] Bouchard DR Dionne IJ Brochu M. Sarcopenic/obesity and physical capacity in older men and women: data from the Nutrition as a Determinant of Successful Aging (NuAge)-the Quebec longitudinal Study. Obesity. (2009) 17:2082–8. 10.1038/oby.2009.109 19373219

[154] Visser M Pahor M Tylavsky F Kritchevsky SB Cauley JA Newman AB. One- and two-year change in body composition as measured by DXA in a population-based cohort of older men and women. J Appl Physiol. (2003) 94:2368–74. 10.1152/japplphysiol.00124.2002 12598481

[155] Santanasto AJ Glynn NW Newman MA Taylor CA Brooks MM Goodpaster BH. Impact of weight loss on physical function with changes in strength, muscle mass, and muscle fat infiltration in overweight to moderately obese older adults: a randomized clinical trial. J Obes. (2011) 2011:1–10. 10.1155/2011/516576 20953373 PMC2952914

[156] Newman AB Kupelian V Visser M Simonsick EM Goodpaster BH Kritchevsky SB. Strength, but not muscle mass, is associated with mortality in the health, aging and body composition study cohort. J Gerontol Series A Biol Sci Med Sci. (2006) 61:72–7. 10.1093/gerona/61.1.72 16456196

